# Review Department

**Published:** 1889-01

**Authors:** 


### REVIEW DEPARTMENT.

THE EAR AND ITS DISEASES, BEING PRACTICAL CONTRIBUTIONS TO THE STUDY OF OTOLOGY. BY SAMUEL SEXTON, M. D., AURAL SURGEON TO THE NEW YORK EYE AND EAR INFIRMARY, ETC. EDITED BY CHRISTOPHER J. COLES, M. D. NEW YORK, WILLIAM WOOD & CO., 1888.

Dr. Sexton is a man of an eminently practical and observant turn of mind. No fact is too minute to escape his notice and he possesses the happy facility of pressing into service whatever he observes. Whilst his work on the diseases of the ear is by no means exhaustive and falls short of covering the whole ground of the subject, it is delightfully desultory and gossipy. He seems to have given free rein to his fancy in the plan of his contributions and to wander at his own free will over the inviting field of otology. Hence he has dealt somewhat lightly with the drier and more technical aspects of his subject and has given to his work a more popular tone and cast. It does not by any means follow from this that Dr. Sexton is not a master of his subject, or that he is incompetent to treat it in its entirety. Such a supposition is disproved by every page of his book which bristles with that suggestiveness that is born of consummate knowledge.

But for his own reasons the Doctor has preferred to make his book what it is rather than a systematic treatise. Doctor Sexton's style is easy and graceful and as limpid as a crystal stream, but the very ease with which he uses his pen betrays him at times into loose and awkward constructions. Among the few such faulty grammatical structures we may instance the following sentence on p. 21 : "Autophonia is common to diseases affecting both the external and middle ear, yet often *differs sufficiently to very much facilitate making a differential diagnosis.*" And again what sentence could be more confused than the following on p. 71 ? "The origin and continuance of aural disease *is* due to or associated intimately with catarrhal inflmmation of the upper air passages in the greater number of instances ; but acute and chronic aural catarrh, constituting the principal affections of the ear, *may be isotated*, (like small-pox ?) *or more or less independent of the former.*" In the very next sentence we find the following strange form of speech : "It has not been long since inflammation of the mucous tract of the head was simply known as "a gathering" in the head. Why not have said "It is not long since, etc." ? A few more sentences of a similar slip shod character might be pointed out here and there throughout the book. But it is not our purpose to be carping or captious and these are smaller matters for criticism. The first part of Dr. Sexton's work is devoted to a consideration of the anatomy and physiology of the ear and contains nothing beyond what is found in the ordinary manuals on the subject. The second part is concerned with the causes of ear disease and contains much that is, if not entirely new, at least highly interesting and practical. The two chapters on "Catarrh of the upper air tract" and on "Oral Irritation" will amply repay perusal. In an admirable diagram the author has pointed out the close and unbroken connection that exists between all the mucus lined cavities of the head and the consequent liability of a catarrhal inflammaticn to spread from one to another. That diseases of the ear should, therefore, spring from catarrh or any portion of the upper air tract is not to be wondered at. The author proceeds afterwards to consider the conditions which tend to produce and foster these morbid states and discusses with interest and knowledge the peculiarities of city and country life as viewed from their bearing on catarrh. He affirms with good reason that our absurd practice of overheating public buildings has much to do with the production of this malady, and that many persons can lay the initial stage of their deafness—a deafness ensuing upon catarrh—to sudden changes from overheated churches, theatres, public halls and school houses, to the chilly air of the street. Dr. Sexton's observation concerning a phase of catarrh witnessed in rural districts is well worthy of consideration. It is so much the vogue to lay at the door of that bugbear "malaria" the thousand and one maladies from which country persons suffer, that it is well to point out to our country medical friends that many nervous disorders, and notably catarrhal influenzas, simulate all the symptoms of malaria. A closer observation of facts has led our author, and many more besides him, to the conclusion that catarrhal troubles of the upper air tract are responsible to a greater degree for the whole train of symptoms that are usually embraced under the head of malarial disease, than is the enchanted denizen of the fens and the marshes. The influence of meteorological conditions in the production of catarrh is strange and subtle. Some persons are so susceptible to these influences that in the presence of an atmosphere surcharged with oxygen they experience unmistakable symptoms of nervous depressions, while the majority of persons experience a feeling of exhilaration. Indeed there is no disease which is calculated to occur amid a more varying set of conditions than catarrh, and this is the impression which is left on the reader's mind after the perusal of this interesting chapter of Doctor Sexton's work. Fortunately the susceptibility is confined to the smaller number of those whose nervous system is abnormally sensitive to the operation of external conditions.

At first glance one would be loth to believe that irritation of the buccal cavity is apt to give rise to aural difficulties, but Dr. Sexton's chapter on "oral irritation" will speedily remove any doubt on this point. Indeed to the anatomist such a consequence would appear natural and almost necessary, for the nervous relationship between the two regions is exceedingly close and extensive. Some of the most protracted and intractable cases of acute purulent inflammation of the middle ear, the author tells us, he has seen associated with the cutting of a wisdom tooth, the duplex dental irritation keeping up the aural trouble. Of course the practical inference from this dependence of a sound condition of the organ of hearing on a faultless condition of the teeth is obvious and should direct early and assiduous attention to the proper care of the latter. The author's observations on the pernicious practice, recommended by so many practitioners, of employing the nasal douche and spray and the post nasal syringe, are deserving of serious consideration. We do not condemn the practice absolutely, since conditions occur no doubt where the advantages of these proceedings outweigh any possible injury that may attend their employment. But as a rule they do more harm than good, and should not be indiscriminately recommended by physicians. The practice of plunging into water is an excellent one *per se*, and much preferable for human beings to the lazy immersion of the hippopotamus, but we should learn a lesson from the procedure of the latter and add some measures for the protection of the ears againt the injuries that the ear-drum is apt to sustain from sudden and violent contact with so dense a medium as water.

We will close this brief and hurried review of Dr. Sexton's excellent contribution to aural science with a reference to his chapter on the education oj school children with defective hearing. Where we number our school children by the hundreds of thousands, and when we reflect, at the same time, that the utmost inequality in hearing capacity exists among them, it is evident that the system of teaching which makes no account of such inequality is radically and essentially defective. How can children whose hearing is impaired by the numberless causes that produce partial deafness be expected to learn where the recitations are carried on in a key and at a pitch suited only to ears of normal power? Dr. Sexton's suggestion that scholars be graded in proportion to this capacity is a most wise one, as are also his indicated precautions against the dangers that result from imperfectly ventilated and badly heated school buildings The close relationship that exists between the ear and brain, and the liability of persons suffering of otitis to sustain cerebral lesions, is also a point of the utmost practical interest and should lead parents and teachers to respect and protect the organ of hearing to a greater extent than they are accustomed to do. Dr. Sexton's book will be read with interest and profit by all classes of persons, and there is no question as to the lasting and substantial results likely to flow from its close perusal. C. M. O'L.

---

LAMENESS OF HORSES AND DISEASES OF THE LOCOMOTORY APPARATUS. BY A. LIAUTARD, M. D., V. M. New, York, W. R. Jenkins, 1888.—As the science of medicine progresses it is found that the special subjects develop so rapidly that they require separate and particular attention from investigator and writer, hence the *raison d' etre* of this valuable little book.

Not since Percivall wrote on lameness some years ago has such an excellent treatise on the subject appeared as this by Dr. Liautard.

Stress need not be laid on the importance of a correct knowledge of lameness as everyone knows as well as the veterinarian how frequent the affections of the extremities are and how serious the consequences, a lame horse being about as reliable as a dead one, or, as the old saying goes, "no feet no horse." It is the commonest disease the practitioner will be called upon to treat, and on the results of his treatment will depend in a great measure his professional success and good name.

The author does not claim that the work contains startling discoveries or new theories, but simply that it is a "contribution of comparatively recent discoveries and a help to a further practical discussion of an important topic."

The book is written in a clear and concise style, and is essentially practical, containing as much valuable matter in its three hundred pages as will be found in many a more imposing volume. Particular attention is given to the rules for the examination of the feet and to the details of every step in the process.

*Chapter I* defines lameness, shows the general causes, symptoms and means of diagnosis. Page 31 should have been in italics— * * * "practitioners are quite too apt to commit the error of overlooking the examination of the foot, looking upon it as a matter of secondary importance and attending to it as a routine and formal affair only. The veterinarian should attend personally to examination of the foot. Every step of the process should be supervised, including the removal of the shoe, the paring of the hoof, if necessary, the exploration with the nippers," * * * "he should never be influenced by the circumstance of a previous examination of a horse shoer."

*Chapter II* takes up the diseases of bones, periostitis, ostitis, osteomyelitis, the diseases of the joints, of muscles, and of tendons.

*Chapter III* treats of the ailments of special localities. The article on shoulder joint lameness is specially noteworthy, as again that upon the diseases of the hock.

If the reader will appreciate the charming satire of Chapter IV on Diseases of the Foot, he will already be on the highway to an understanding of these troubles better than is commonly derived from the ordinary classification. Diseases of the foot are classified as Paronychia, in five groups :—P. Ungualis, P. Cellulosa, P. Tendinosa, P. Cartilaginosa and P. Osseosa.

We heartily welcome this book as a valuable contribution to the Veterinary literature of America. J. O'L.

---

NATURE'S HYGIENE: a systematic manual of natural hygiene, containing a detailed account of Eucalyptus, Pine and Camphor forests and industries connected therewith, by C. T. Kingzett, F. I. C. London: Balliere, Tindall & Co. 1888.

This work of Mr. Kingzett reveals a new departure in chemical science and will be read with as much interest by specialists in chemistry as by the intelligent lay reader, since it is a pure and simple unfolding of a page in the great book of nature. The author leaves the pent-up atmosphere of the laboratory, and walking forth into the field and the forest, points out to us the marvellous and efficacious antidote to disease which the beneficent mother everywhere supplies with a most lavish hand. We smell the freshness of the woodland in Mr. Kingzett's book, and we inhale the balmy air of the incense laden pineries of the North and the boundless stretches of the Australian Eucalyptus forests throughout its pages. The work possesses the sort of practical interest that recommends it equally to the official Sanitarian, the operative Surgeon, and the general practitioner of medicine. The chapter on "Bioplasm and its Functions" is highly interesting from the evolutionist's point of view; but it is especially in the chapters devoted to he comparative study of antiseptics and disinfectants that the general reader will take pleasure. The efficacy of the hydroxye or peroxide of Hydrogen as an antiseptic is fully established, as well as the general adaptability of the "Sanitas" preparations to the same purposes as those for which carbolic and salicylic acid are used at present. These marvellous products of the oxidisation of the oil of turpentine have been shown to possess the power of arresting every species of fermentation with rapidity, and yet being entirely free from poisonous properties. Their use is now universal throughout the hospitals of Great Britain, and such, we presume, will soon be the case in this country. C. M. O'L.

TRATTATO DI TECNICA E TERAPEUTICA CHIRURGICA DEGLI ANAMALI DOMESTICA DEL DOTT N. LANZILLOTTI BUONSANTI, DIRETTORE DELLA SCUOLA VETER. DI MILANO. Vol. I. Milano: Fratelli Dumdland. 1889.

Quite a number of works on Veterinary Surgery have been recently published, notably those by Fleming, Vogel, Degive, Peuch & Toussant, etc.; all excellent text-books, but to judge by the part received, volume I., the one under consideration is far in advance of them. For its methodical arrangement, novelty of material, number of excellent illustrations, which, though roughly executed, have the merit of originality, this work deserves to be classed among the practical text-books which every Veterinary student and practitioner should possess.

This new publication has two volumes, the first, of 600 pages, being devoted to General Surgery; and the second, which is not yet published, to Special Surgery.

The treatment of wounds, disinfection, drainage, etc., receive special attention, as also, the hygiene and treatment of the animals operated on, anæsthesia, and the accidents resulting therefrom, methods of securing animals for operation and bandaging. Bloodletting and the transfusion of blood are carefully treated.

In all operations the method of Prof. Lister is the one most highly recommended.

Hydrotherapy, massage, and the application of electricity, have each a chapter devoted to them,—and one each to vaccination and inoculation.

J. O. L.

THE PHYSICIANS' VISITING LIST FOR 1889. P. Blakiston, Son & Co., Philadelphia.

This handy visiting book is now in its thirty-eighth year of publication. It contains, besides a dose table, a list of new remedies, together with many other references handy for a physician.

